# Normalisation and equity of referral to the NHS Low Calorie Diet programme pilot; a qualitative evaluation of the experiences of health care staff

**DOI:** 10.1186/s12889-023-17526-2

**Published:** 2024-01-11

**Authors:** Kevin J. Drew, Catherine Homer, Duncan Radley, Susan Jones, Charlotte Freeman, Chirag Bakhai, Louisa Ells

**Affiliations:** 1 Obesity Institute, School of Health, Leeds Beckett University, City Campus, Leeds, LS6 3QW UK; 2 Sport and Physical Activity Research Centre, Sheffield Hallam University, Olympic Legacy Park, 2 Old Hall Road, Sheffield, S9 3TU UK; 3 Obesity Institute, School of Sport, Leeds Beckett University, Headingley Campus, Leeds, LS6 3QW UK; 4 School of Health and Life Sciences, Teesside University, Middlesbrough, Tees Valley TS1 3BX UK; 5 Public Health Calderdale Metropolitan Borough Council, Halifax, HX1 1TS UK; 6 Larkside Practice, Churchfield Medical Centre, 322 Crawley Green Road, Luton, Bedfordshire LU2 9SB UK

**Keywords:** Type 2 diabetes, Obesity, Low calorie diet, Equity, Inequalities, Normalisation process theory, Re:Mission study

## Abstract

**Background:**

Health and wellbeing can be profoundly impacted by both obesity and type 2 diabetes, while the normalisation and equity of care for people living with these non-communicable diseases remain as challenges for local health systems. The National Health Service Low Calorie Diet programme in England, aims to support people to achieve type 2 diabetes remission, while also reducing health inequalities. We have explored the experiences of health care staff who have made a referral to the LCD programme, while identifying effective and equitable delivery of programme referrals, and their normalisation into routine care.

**Methods:**

Nineteen individual semi-structured interviews were completed health care staff in the first year of the Low Calorie Diet programme. Interviewees were purposively sampled from the ten localities who undertook the Low Calorie Diet programme pilot. Each interview explored a number of topics of interest including communication and training, referrals, equity, and demands on primary care, before being subjected to a thematic analysis.

**Results:**

From the data, five core themes were identified: Covid-19 and the demands on primary care, the expertise and knowledge of referrers, patient identification and the referral process, barriers to referrals and who gets referred to the NHS LCD programme. Our findings demonstrate the variation in the real world settings of a national diabetes programme. It highlights the challenge of COVID-19 for health care staff, whereby the increased workload of referrals occurred at a time when capacity was curtailed. We have also identified several barriers to referral and have shown that referrals had not yet been normalised into routine care at the point of data collection. We also raise issues of equity in the referral process, as not all eligible people are informed about the programme.

**Conclusions:**

Referral generation had not yet been consistently normalised into routine care, yet our findings suggest that the LCD programme runs the risk of normalising an inequitable referral process. Inequalities remain a significant challenge, and the adoption of an equitable referral process, normalised at a service delivery level, has the capacity to contribute to the improvement of health inequalities.

**Supplementary Information:**

The online version contains supplementary material available at 10.1186/s12889-023-17526-2.

## Introduction

 Health and wellbeing can be profoundly impacted by obesity and type 2 diabetes (T2D), which are both prevalent non-communicable diseases [1]. In England, 26% of adults live with obesity [2], while 3.2 million adults are known to live with T2D. Modelled projections indicate an escalation in the costs associated with obesity and T2D, to the National Health Service (NHS) and wider society, unless urgent action is taken [3]. Recent systematic reviews [4–8] and clinical trials [9–11] have shown that for some people living with, or at risk of obesity and T2D, a low calorie diet achieved by Total Diet Replacement (TDR), can lead to clinically significant weight loss, support remission of T2D, and improve quality of life, and that such approaches can be cost-effective [12, 13]. However, it remains a continued and significant challenge to translate the findings from clinical trials into routine service delivery in the real-world settings of local health systems (primary care in the UK).

Issues relating to normalisation remain pertinent to the delivery of interventions across primary care, and the extent to which a new practice is routinised is taken as an indicator of success of implementation processes. Normalisation is regarded as the repeated actions of individuals who engage with some ensemble of activities to a point at which it becomes routinely embedded in any given cultural, social, and/or political context [14]. The normalisation of referrals to interventions must therefore occur in already existing and socially patterned knowledge and practices embedded within primary care settings. Normalisation Process Theory (NPT), which has been used to interrogate and understand processes of normalisation and has been widely used within implementation science [15] (see also example from current study (Jones et al.: Commercial provider staff experiences of the NHS Low Calorie Diet programme pilot: a qualitative exploration of key barriers and facilitators, submitted)), consists of four mechanisms; coherence, cognitive participation, collective action and reflexive monitoring. These mechanisms are centred on the social organisation of work needed to ensure new practices become routine and sustained over time [14].

Furthermore, addressing health inequalities, the unjust and avoidable differences in people’s health outcomes, has also proved a continued and significant challenge within health care systems despite being a public health priority [16]. For example, the prevalence of both obesity and T2D increases with area-level deprivation, and amongst people of Black and South Asian ethnicity [17, 18]. Yet, interventions aimed at improving health across the entire population can be markedly more beneficial for individuals of higher socio-economic status, and of White ethnicity [19–22], which results in the occurrence of intervention-generated inequalities. However, inequalities also exist in access to healthcare. For example in England, completion of the recommended annual checks for people living with diabetes are lower in those living in more socioeconomically deprived areas [23]. Importantly, inequalities are created, perpetuated and exacerbated by inequities, thus, the state in which people do not have a fair and just opportunity to access health care is also regarded as an inequity that perpetuates the higher prevalence of obesity and T2D in certain populations [16, 24]. To address this inequity, it is argued that there is a need for work to achieve greater equity at varying levels. These include at a national or policy level, and as we have discussed elsewhere [25], at an organisational or planning level (local health systems). However, the service delivery level, where health care staff make patient referrals, is deemed equally important [26–30].

For people seeking health care for T2D and obesity, health care staff within primary care play a critical role as they operate as the primary point of contact. Thus, health care staff, such as physicians and nurses, and their practices and engagement with individuals, directly contribute to the opportunities and experiences those individuals have, and the subsequent relative success of treatment options pursued [31–37]. For example, health care workers have been shown to hold weight-biased attitudes toward people living with obesity which can impact directly upon the provision of care [38]. Thus, better understanding of the roles and behaviours of health care staff can raise the standard of care and improve outcomes, while also allowing for a greater understanding of the normalisation of, and inequalities in, access to health care.

### The NHS Low Calorie Diet programme pilot

A national Low Calorie Diet (LCD) Programme, available to adults (18–65 years) with a Body Mass Index (BMI) ≥ 27 kg/m² (adjusted to ≥ 25 kg/m² for Black, Asian and other ethnic groups) and a T2D diagnosis within the previous 6 years (full eligibility criteria [39]), was launched in England in September 2020. Commissioned by NHS England in partnership with Diabetes UK, the NHS LCD Programme (as of June 2023, renamed the NHS Type 2 Diabetes Path to Remission Programme) was delivered across ten integrated care systems^1^ [40], selected for their geographical and socio-demographic diversity (referred to hereon in as localities) (see Additional file 1).

Each locality tested one of three different delivery models (group, 1:1 and digital), whilst each delivery model consisted of three distinct phases, including TDR (12 weeks), food reintroduction (4–6 weeks) and maintenance (34–36 weeks). During the TDR phase, commercial providers provided TDR products at no cost to service users. The product brands and range varied, for example, one provider provided six options of soups and shakes, while another provider provided 89 options of soups, shakes, smoothies, bars, breakfasts, and pre-prepared meals. All service users were instructed to take four TDR products each day, which amounted to between 800 and 900 calories during the first phase of the programme.

The fulfilment of a commitment made in the NHS Long-Term Plan [41], the LCD Programme aimed to improve the health of eligible individuals while also aiming to reduce health inequalities, and associated future costs to the NHS. Delivered by commercial providers, the LCD programme was reliant on the referral of eligible people from primary care, of which it had received 7,554 by December 2022 [42]. This paper, therefore, aims to explore the experiences of health care staff who have made a referral to the LCD programme, while identifying effective and equitable delivery of programme referrals, and their normalisation into routine care.

## Methods

This study received ethical approval from the Health Research Authority (REF 21/WM/0136), and is reported using COREQ guidelines (see Additional file 2) [43]. Using a purposive sample, health care staff with experience of patient referral to the LCD programme (referred to hereon in as ‘referrers’), were recruited equally across the first ten localities who undertook the programme pilot. Maximum variation in the sample was achieved by recruiting one high- and one low-referrer from each pilot area, as defined by local health service leads, who assisted the recruitment process. In total, nineteen referrers (14 females and 5 males) with experience of referring to the programme and employed within GP practices (as either Practice Nurses x10, General Practitioners x6, Clinical Pharmacists x2 or Advanced Nurse Practitioners x1) were interviewed. Four interviewees were also undertaking roles as clinical leads for the LCD programme in their localities at the time of the interview, one of which had not made a referral but had overseen the process in their locality.

Semi-structured interviews (MS Teams) lasting between 25 and 40 min were carried out by two researchers (KD and CF), each conducting seven and 12 interviews respectively, between September 2021 and April 2022. An interview guide was designed to elicit discussion on specific topics of interest, and was piloted by the research team before being communicated to interviewees prior to interview. Topics included: communication and training, referrals, equity, and demands on primary care, and were pre-empted by initial programme theory [44], developed through the overarching realist informed Re:Mission evaluation [45]. Fieldnotes were recorded after each interview.

Interviews were audio recorded, transcribed verbatim, and then read multiple times by KD and CF who familiarised themselves with the data before being subjected to a thematic analysis as described by Braun et al. [46]. Using a latent coding method, transcripts were coded using the interview guide as a deductive framework, ensuring the realist-informed evaluation underpinned analysis. This involved the mixing of inductive and deductive reasoning which facilitated movement between participant accounts and researcher defined topics of interest. For example, communication and training were selected by the authors as topics of interest given their relevance to the rollout of the LCD programme. However, processes of inductive reasoning meant that what participants collectively said about communication and training led to the construction of sub-themes which subsequently provides the detail for the pre-emptively selected theme outline. NVivo software (QS International Pty Ltd. Version 12) was used to assist the storing and organising of textual data and initial coding.

The use of thematic analysis allowed for the identification of patterns (‘themes’) in the data. The initial identification and reviewing of themes were conducted by CF. These themes were then subjected to a further interrogation by KD, who read through and coded all transcripts to search for alternative meanings in the data not previously tagged, before further reviewing, defining, and naming themes to consolidate themes into clusters. Clusters, that capture more than one specific idea, allowed what Braun et al. call ‘higher-level’ patterns in the data to be identified. A third researcher (CH) reviewed the resultant thematic report, which led to the refinement and consolidation of themes and the development of recommendations.

## Findings

Upon completion of the analysis, five core themes were constructed out of the data. The following section presents these themes, along with exemplar quotes, whilst further supporting quotations can be found in Additional file 3.

### COVID-19 and the demands on primary care (theme 1)

The LCD programme pilot was first launched in September 2020, following postponement from April 2020 due to the COVID-19 pandemic. During interviews, many referrers discussed GP practices being overburdened as they were “*bombarded*” (R2 – Advanced Nurse Practitioner) with work and dealing with a “*tsunami of patients*” (R9 - GP), who were said to have suffered negative consequences as a result of the pandemic. A shortage of time, discussed more frequently by GPs than practice nurses, was a key contributing factor to referrers feeling overburdened.

*“I think our limitation has been the time and also the NHS work pattern at the moment in terms of the demands, in terms of the vaccinations and in terms of the increased COVID cases and the respiratory infection rates, etc” (R13 - GP).*

A lack of capacity in primary care, perpetuated by staffing shortages, was also an issue. Referrers discussed both the redeployment of staff onto Covid related work, such as the vaccine rollout, as well as a higher than normal turnover of staff during this period. This all contributed to a sense that interviewees were working under increased pressures and dealing with delays and a backlog brought about by COVID-19.

### The expertise and knowledge of referrers (theme 2)

Referrers, who made between 0 and 38 referrals each, generally heard about the programme via written communication, typically via e-mail or newsletters and bulletins. Half of referrers, some of whom were the practice diabetes leads, discussed completing LCD specific training, either by attending a workshop remotely or in person, or by watching a recording online. A smaller group did not attend training, either because training was not available in their locality, it was deemed unnecessary, or it was something that their practice diabetes leads attended before relaying information on to them.

*“I wasn’t on the initial training about the programme, a handful of the GPs were. But one of the partner GPs, she’s the diabetes kind of lead, she’s the one I discuss mostly with and she told me about it first” (R12 - Practice Nurse).*

Half of the referrers interviewed said the information they received about the programme was appropriate, easy to follow, and straight forward, thus facilitating the referral process. Furthermore, a number of referral staff, including those who acted as local clinical leads for the LCD programme and had responsibility for delivering LCD training, discussed already having appropriate knowledge about diabetes and the LCD programme more generally.

Some referrers, most of whom were practice nurses, discussed not receiving all the information they would have liked about the programme, including details about the patient journey and aspects of the programme pilot (i.e., the timing of phases, the delivery model in their locality, the rescue package).

*“when I refer my patients to Desmond*^2^, *[…] I know exactly what they’re going to experience on that day or the two day programme because I went along as a health care professional to see what they’re being taught. […] Whereas with the low calorie diet, I don’t have that information” (R6 - Practice Nurse).*

Other referrers wanted more information about the eligibility criteria, particularly factors relating to ineligibility, and the biological effects and challenges of weight loss. One referrer alluded to the need for referrers to have greater cultural competences when referring people from minority groups.

*“it has to be recognised that you’ve got communities that are, have a different culture and we need to […] put that into…. So, for example potentially having not just an interpreter, but somebody who very much knows about that culture being the one that will be talking to that person” (R14 – Pharmacist).*

A number of referrers also discussed needing greater support, or “*a little bit of a push*” (R8 - Practice Nurse) to refer either by having an engaged locality lead or clinical lead that maintained contact throughout the year, or by having a sense of how people were getting on once referred.

*“I’d really like to know some success stories because they will help me tell people how, how good it’s been when I’m talking about it as an option” (R12 - Practice Nurse).*

Half of referrers, mainly practice nurses and pharmacists, discussed generally having limited knowledge of how those referred were progressing on the programme, which both led to some uncertainty about the care needs of individual patients as well as about the programme more broadly.

### Patient identification and the referral process (theme 3)

To identify eligible patients, referrers discussed running system searches in their practices, either by creating their own searches or by using a search shared by the provider or colleagues in the local health system. Searches essentially created lists of potentially eligible patients, who were then either contacted by text, letter or in one case, a telephone call, to arrange one-to-one telephone consultations. This proactive identification of patients, carried out by just over half of referrers, was seen by some as a preferable approach.

*“We contacted them. We didn’t wait for the annual review because sooner is better. […] Because you get this clinical inertia. We wait all the time, but we know that when we wait, there’s nothing to gain” (R6 - Practice Nurse).*

However, it was not an approach that all referrers adopted, either due to a lack of capacity, or because their locality had allocated their practice a small number of places. Furthermore, of those that did run a search, many only did so at the outset of the LCD programme.

*“We did a search and there were nearly 200 patients, that took me some time. I was just planning to ring the patients to book appointments but, in the meantime, I discussed [it with colleagues] and I mentioned that we had to find a couple of patients to refer. Within the week without any difficulty, it was so easy because then patients came for health checks and chronic disease review. I was immediately sent five patients, so I contacted them, and they agreed. So, it was quite an easy process. I didn’t need to go through my 200 patient list at all, then I started referring opportunistically, when they came for healthcare checks and chronic disease review” (R17 - GP).*

Indeed, all referrers discussed referring people to the programme opportunistically, or “*by chance*” (R15 - Practice Nurse). This involved discussing the programme as an option during other appointments, most notably during diabetes reviews, which were often seen as “*the best time to get people*” (R19 - Practice Nurse) to consider the programme.

*“It’s either the patient already knows about the programme and then presents to the GP surgery or it’s opportunistic. In my particular practice it’s opportunistic, mentioning the programme or offering the programme with a patient and discussing it as part of their diabetes review” (R1 - GP).*

Subsequently, half of referrers discussed the “*more health conscious*” (R10 - Pharmacist), or those already engaged in their care as the people most likely to access the programme.

*“I think that there’s a, there’s a well kind of documented issue amongst kind of that proactive care that if we don’t go looking for that particular patient group, the people who tend to engage tend to be the, the more engaged with their health, they tend to be less subject to inequalities anyway, so actually the kind of people who we opportunistically check are, tend to be the slightly more engaged patient groups anyway. So, my concern would be that by being opportunistic in terms of bringing up it, you know bringing things up like this in a review automatically we know that that person a) is attending for their review in the first place, but also has had the previous tests that have let them get to that point” (R1 - GP).*

Six referrers also discussed their active decisions underpinning patient identification, either by means of working through lists of potentially eligible people or by how diabetes reviews were being scheduled. Some relied on dates, such as calling people based on their month of birth, or by prioritising people who were newly diagnosed. One referrer discussed “*looking at [people with] the high BMI*” (R8 - Practice Nurse) as a means of identifying people most in need. Others relied on more arbitrary approaches, such as already knowing who might be interested, or by operating on a first-come first-served basis.

*“it was a first come, first served basis. […] I identified around 21 because they had to be diabetic in the last six years and needed to fit certain criteria. […] Within a week I think I had hit seven [referrals]” (R4 - GP).*

Once people had been identified, the majority of referrers positively discussed the referral process, which was said to be “*quick*” (R8 - Practice Nurse), “*easy and straightforward*” (R17 - GP) and “*fine if you meet all the criteria*” (R3 - Practice Nurse). The referral form, used differently across the ten localities, was discussed as a key part of the referral process. Usually embedded within the practices’ information systems and auto populating with relevant data, the referral form helped prompt referrers, and essentially guided them through the referral process.

*“It’s a good process […] because it’s all in black and white. The form is really good because it helps remind you […] everything on the form” (R13 - GP)*.

*We’ve got a very useful template which maybe articulates what you need to do for the referral so that’s very useful. It’s easily emailed using our AccuRx which is useful […] so that means that I can email straight away during the consultation rather than having to ask somebody else to email which happens for some outside organisations. So, I would say the useful template, a fairly quick and easy form, makes things easy” (R2 - Advanced Nurse Practitioner).*

However, referrers also discussed having to go through a period of learning regarding the referral process. Described as “*a learning curve*” (R11 – Practice Nurse), referrers discussed submitting incomplete referrals, before the process became “*quicker*” (R18 - Practice Nurse) and “*part of the conversation*” (R7 - Practice Nurse) with patients.

*“If you’d spoken to me at the beginning my answer would have been very different. I was quite frustrated initially because it seemed like every referral I sent got sent back to me. And obviously it took time to do all the referrals and we had a list of patients that wanted to be referred” (R11 – Practice Nurse).*

### Barriers to referral (theme 4)

Barriers associated with the referral process were highlighted, of which the workload of referring to the programme was discussed most frequently. The majority of referrers suggested the referral process represented additional workload. This was framed by some suggestions that referring to the LCD programme was not “*core work*” (R2 - Advanced Nurse Practitioner) and therefore making referrals was not prioritised if the additional workload could not be accommodated. Running searches and the subsequent work to invite eligible people was discussed as time consuming, and thus not carried out by all referrers. The process of referral was also regarded as time consuming, although about a third of referrers, the majority of whom were practice nurses and pharmacists, felt that the time required did not “*make the job harder*” (R3 - Practice Nurse).

*“I think the only thing I’ve found is it’s quite time-consuming. So, although we know how beneficial it can be, and we really want to promote the programme and to get patients onto the programme, it is quite time consuming. So, we send the letter out, so I do the audit, send the letter out to the patient and then they book a telephone call with myself and then if the patient was on blood pressure medication and needed to see a prescriber, I’d then have to ask the nominated GP to give the patient a call to discuss stopping that medication on the first day of starting the programme” (R5 - Practice Nurse).*

Issues of workload and time meant that for some referrers, certain roles in primary care were better placed to discuss the programme with people who may be eligible.

*“It’s probably a little bit better for Specialist Pharmacists or Specialist Nurses whose clinics are a little bit [longer] …the GPs, I think they don’t have that time.” (R14 - Pharmacist).*

The need to agree medication changes, most commonly deprescribing, in relation to referrals was considered to result in additional workload and was perceived by some to be a “*huge block*” (R9 - GP) as “*GPs are less comfortable with the idea of making medication changes purely from the fact that they don’t do it a lot*” (R1 GP).

*“You don’t need to be quite so scared of the deprescribing. Because that’s been a huge block you know. Certainly, I had a nurse the other day that I said it to last, I think last Wednesday, and it was like “I’m sitting on three referrals, I’m just not sure”. And it’s, it’s about the confidence to do the deprescribing” (R9 - GP).*

Indeed, most referrers acted as programme champions as they were “*really passionate about the Low Calorie Diet*” (R8 - Practice Nurse) and thought the project was “*really important*” (R16 - GP). Thus, the referrer, and their keenness towards the LCD programme and the confidence in the referral process were important elements in the generation of referrals.

A minority of referrers also discussed other barriers associated with the referral process. Referrals were typically opportunistic and conducted during contact points, such as annual reviews; thus, when COVID-19 disrupted routine care delivery, it also led to reduced scope for opportunistic referrals. Also, the programme criteria meant that “*quite a few patients haven’t been eligible*” (R18 – Practice Nurse) for the programme, often due to their age or the duration of T2D diagnosis, was also perceived as a barrier by some referrers. Finally, as discussed elsewhere [25], a number of localities adopted an approach to allocating programme places, which had the effect of constraining GP practices in referral generation. For example, in one locality, GP practices were originally told by the locality lead that they could only refer up to 1% of their eligible population.

*“I created my own search on SystmOne, identified around 20, 21 patients who I could have possibly referred on, but at that time the LCD programme would have only accepted seven or nine from my practice because they were giving it proportionately to all the practices in [Area] CCG. So, I hit the seven within a week” (R4 - GP).*

Perceived barriers were also discussed in relation to the patient population, specifically, why referrers considered that people might decline the programme or, alternatively, progress to starting it. Most notably, half of the referrers discussed the intensity and level of commitment required, noting that some people do not want “*to go on a liquid diet completely*” (R4 - GP).

*“They just don’t want to stop eating food altogether you know for that time because they might have weddings, I had one person say well I’ve got a wedding in a couple of weeks, and I’ve got a christening here and I’m going to a party there and I want to eat a bit of cake and things like that you know. So, I think that stops it a little bit, people don’t want to stop eating for that amount of time […] they don’t want to live on shakes and soups” (R3 - Practice Nurse).*

The events people had planned during the initial 12 weeks of the programme, and a perceived lack of TDR variety were contributing factors to the programme being of limited attractiveness to some people. Furthermore, referrers highlighted that people often wanted to explore other options first, and that some associated the LCD programme with other weight loss approaches they had tried previously without lasting success.

### Who gets referred to the NHS LCD programme (theme 5)

When asked about equity and inequalities in relation to the referral process, referrers discussed who does and does not get referred to the LCD programme. Nearly half of referrers suggested that the referral process did not result in any groups being excluded.

*“We’re trying really hard to refer everybody and I don’t think that we’ve excluded any groups” (R9 - GP).*

However, referrers also reported that remote, and technology-assisted programme delivery could be a barrier for people that did not have access to the technology or to an internet connection. Conversely, these same features of the LCD programme pilot, were a facilitator for groups that might have previously struggled to attend in person.

*“I’ve had a few people who have not been able to go ahead with it because, you know, they’re not computer savvy. There are still a few people out there who don’t have the internet who’ve not been able to do it, so with non-COVID times yes, they would have been better off with like a face to face group sessions or something. But then a lot of people like it done remotely because they can fit it in around work and, and kids and school drop off” (R7 - Practice Nurse).*

Furthermore, the inclusionary and exclusionary impact of technology was discussed as impacting at different points, from the distribution of promotional videos that “*does limit it to people with smart phones*” (R16 - GP), through to the attendance of “*people that wouldn’t have participated in the group courses and would be more likely to do these things remotely*” (R5 - Practice Nurse).

Half of the referrers also discussed how language can become a barrier. Many referrers worked in practices which served diverse communities in which people often spoke limited English and relied on family members or interpreters to access healthcare. Some referrers suggested that “*unless things are delivered in different languages, then I think it’s, that’s always going to be a barrier*” (R2 - Advanced Nurse Practitioner). Indeed, one referrer spoke multiple languages, and referred patients to an Urdu specific group; thus, both the referral process and programme delivery conducted in other languages supported engagement for a community of people who spoke limited English.

*“The consultations go English, Urdu, Punjabi is sort of a dialect of Urdu language so Punjabi. And then we have Bengali as well but for that I need to get another colleague to sit in with me or speak with a family member who speaks English to translate into Bengali” (R10 - Pharmacist).*

*“We’ve got three patients […] that were gonna join the programme from our practice. All those patients though they, their grasp of English is very basic, so if it wasn’t in Urdu, they wouldn’t have joined the programme” (R10 - Pharmacist).*

That said, other people with limited English were able to access the programme with the linguistic support of others, notably younger family members.

Technology and language aside, a small number of referrers also described their perceptions of why people from other cultural contexts might not engage with the programme. Specifically, these referrers perceived the cultural food practices of some groups to be why the LCD programme may not “*suit them at all*” (R6 - Practice Nurse), and why they “*still eat a traditional diet*” (R12 – Practice Nurse).

*“The non-English speaking […]. They don’t take up the very low calorie diet because it’s very difficult to explain to them. And they don’t understand the concept of not eating for 12 weeks […] they will most likely carry on eating […] and then top it up with the sachet or something like that” (R6 - Practice Nurse).*

*“When you introduce or you say well, there is this programme you start with three months […], that kind of gets them, ‘oh no, how do I, you know, I like my chapattis, I like my curries, oh how am I going to go from having that and then just having soups and shakes’” (R14 - Pharmacist).*

A small number of referrers articulated the impact that technology, language, and culturally-informed perceptions had on the referral opportunities that some people from certain backgrounds were offered. Three referrers suggested they “*would probably be less likely to offer it*” (R1 – GP) or “*wouldn’t necessarily invite*” (R12 – Practice Nurse) some people from certain backgrounds to join the LCD programme.

*“I’d see a couple of barriers, firstly there might be a bias that somebody for who, for whom perhaps English isn’t their first language or perhaps are they suffered with multimorbidity or perhaps older or, or recognised as being frail. Then perhaps there’s a bias there that because I know it’s a technology-led intervention, I think personally I would probably be less likely to offer it, which is, which is wrong, but nevertheless, that’s true” (R1 – GP).*

## Discussion

In this paper, which contributes to a larger programme evaluation [45, 47], we have explored the experiences of health care staff within primary care who are responsible for referring eligible people to the LCD programme. At a fundamental level, the variation and often conflicting nature of our findings can be explained, in part, by the significant differences in the cultural, social, and political contexts within the real-world settings in which the programme was being delivered. For example, referrers were sampled from ten diverse geographic and sociodemographic localities, which meant referrers worked with people from different ethnic, cultural and socioeconomic backgrounds, and from different rural and urban settings (see Additional file 1). Finally, as we have discussed elsewhere [25], each of the ten localities mobilised the programme with differing approaches to training, incentivisation, and the management of referrals.

The workload for primary care associated with referring to the LCD programme during the interview period occurred at a time when practices had multiple competing pressures and reduced operational capacity due to the COVID-19 pandemic, which itself had exacerbated health inequalities [48–50]. Whilst this additional workload was more marked for GPs, who often had responsibility for deprescribing medication and thus greater demands in comparison to some other referrers, this finding was consistent across multiple referrer accounts. Furthermore, this occurred in a setting where time was already considered a constraining factor for the management of T2D [34]. As a result, this temporal context, specific to the early period in which the programme was launched and available, was a significant barrier to referrals, and likely had an impact on processes of normalisation.

### Normalisation of referrals to the NHS LCD programme

Using NPT theory as a sensitising lens selectively, as others have done previously [15], we have used the three mechanisms of coherence, cognitive participation, and collective action, to help elucidate and understand processes of normalisation within our findings. Coherence recognises, in the first instance, that referrers have to be able to make sense of the LCD Programme and referral process [51]. Our findings show that coherence was increased for some referrers because they had sufficient expertise and knowledge, while many referrers acted as champions for the programme suggesting levels of acceptance were present. Referrers also reported going through a learning curve before acquiring greater coherence, which in turn appears to have reduced the workload of referring. Thus, there is evidence in our findings that the programme made sense to many referrers. This process of sense-making is necessary in order to promote the routinisation of discussing the programme with individuals and referring them to the LCD programme [51].

Our findings also reveal important threats to coherence, and thus the process of normalisation. Not all referrers considered themselves to have sufficient expertise and knowledge, which impeded their ability to make sense of the programme, as did concerns relating to the deprescription of medication, which has been reported elsewhere as a barrier to engagement on a similar low calorie diet TDR programme [52]. Furthermore, issues of capacity within primary care which led to referral generation not always being prioritised amongst other workload demands, and the differing means of identifying eligible people had potential to threaten coherence. These threats to coherence may constrain normalisation, by means of hindering cognitive participation and collective action.

Cognitive participation, or buy in, is the process that primary care needs to undertake in order to ensure referrers engage with a new practice, while collective action is the subsequent work, or operationalisation, that is needed to enact a new practice, such as referring to the LCD programme [51]. Our findings suggest that while cognitive participation was favourable for many referrers, in others it was somewhat impeded by constrained capacity, limited feedback on patient progress, and gaps in training. Similarly, our findings suggest that while most referrers found the referral process to be easy and straightforward, it took time to learn, effort was needed to overcome barriers, and referrers needed to build up confidence, which posed threats to collective action. The impact of this reduced buy-in and operationalisation of referrals to the LCD programme represented threats to the referral process being normalised into routine practice. Our findings also show examples of cognitive participation and collective action taking place. Buy-in from senior practice staff was evident and had been supported with training for other referrers. Workable referral processes, such as employing opportunistic referrals, were promoted and widely utilised. Thus, these signs of cognitive participation led to collective action, and a number of referrers referred high numbers of people to the LCD programme.

With the highlighted challenges to coherence, it is perhaps unsurprising that overall evidence of cognitive participation and collective action at the time of interviews was mixed. This is important because the buy-in and operationalisation of new practices rely, to an extent, on referrers making sense of that new practice. Yet, given the disrupting influence of COVID-19 and the broad variation in cultural, social, and political contexts of the ten localities, our findings suggest that the process of achieving coherence, and thus cognitive participation and collective action, was still ongoing at the point of data collection. Indeed, the majority of interviews occurred only one year following programme launch. Despite this, threats to coherence ultimately have the capacity to constrain and undermine normalisation and, at the point of data collection, referrals to the LCD programme were not yet consistently normalised into routine care.

### Inequity at the point of referral

Health equity, the state in which people have a fair and just opportunity to attain their full health and wellbeing, irrespective of their social position [24], is an important consideration for interventions that seek to address health inequalities. Our findings show that referrals to the LCD programme, despite the well-meaning intentions of referrers, are at risk of being inequitable. The principal process of identifying people for referral, adopted across all 10 localities, is to identify people opportunistically; thus, people already engaged in care are given precedence. Furthermore, utilising a first-come first-served basis for offering referral, as was the case in several localities, accentuates potential inequity as those more readily attending primary care are more likely to be referred.

The structure of the referral process was not, however, the only source of inequity at the point of referral. Remote and technology-assisted programme delivery, as well as language, could be barriers to referrals. Language has been previously reported as a significant barrier to accessing weight management services by ethnic minorities, while people living in deprived neighbourhoods have a disproportionate lack of access to digital services [53]. Consequently, the National Institute for Care and Excellence recommend that the design and delivery of weight management is approached with sensitivity to the languages spoken by those the intervention is intended for [54]. Indeed, our findings show that when a referrer has the capacity to speak the same language as people eligible for the programme, this acts as a facilitator for making referrals. However, appropriate language provision on its own may be insufficient to ensure equity if other issues are not also addressed.

Our findings also show that a small number of referrers described their perceptions of why people from other cultural contexts might not engage with the programme. These perceptions appeared to exist outside of any immediate and conscious awareness but could act to unfairly restrict opportunities to access the programme and thus introduce inequity at the point of referral on the basis of a demographic characteristic. Regarded as a subconscious bias, these negative associations, formed on the basis of a characteristic deemed irrelevant, have been reported to affect the decisions health care workers make [55]. Referrers, therefore, appear to have subconsciously allowed their perceptions of patients from ethnically diverse backgrounds to influence the care they provide; thus, a patient’s ethnicity had an impact on the opportunities presented to them to attain health and wellbeing, which appears to be unfair and unjust.

We contend that the perceptions of reduced suitability for people from certain cultural contexts tended to be multifactorial and connected to other concurrent barriers, such as their lack of English speaking and/or access to technology. Thus, the subconscious bias of referrers emerged in contexts where people from minority ethnic backgrounds faced other barriers. In a temporal context where capacity in primary care was acutely limited, this small number of referrers may have perceived certain referrals to be too great a challenge and did not appear to fully recognise the social determinants of health, which for certain individuals can cumulate to create structural disadvantage. These have previously been reported to be somewhat unrecognised, in part because of a tendency for greater focus in primary care on clinical or behavioural determinants of health [30, 56]. There therefore exists the risk that an opportunistic approach to referral generation, in combination with other barriers to the identification of suitability, may result in unjust and avoidable differences in health outcomes, given that inequalities in health outcomes are created, perpetuated, and exacerbated by inequities in access to health care. The LCD programme, which aims to reduce health inequalities, could therefore lead to intervention-generated inequalities if there is inequity at the point of referral (a so-called inequality paradox [57]).

### Limitations

This is the first study to explore the experiences of referral staff with the job of referring patients to a national LCD programme in real-world settings. However, we have identified several limitations: 1) The programme was mobilised in the middle of the Covid-19 pandemic, which itself placed significant strain on the health system and will have undoubtedly impacted upon our recruitment of referral staff, and the processes undertaken by the referrers we did recruit. 2) There were no notable differences expressed by high- and low-referrers, which is likely to reflect how local health service leads defined what high and low meant to them locally. We have previously written about how certain localities initially adopted referral allocations [25], which will have meant some referrers were unable to refer, for example, more than three patients, which in comparison to areas that relied on a small number of referral-generating practices could be considered low. 3) We did not record the demographic characteristics of referrers and thus have not reported them here. However, considering our findings, the demographics characteristics of referrers, such as their ethnic and cultural backgrounds, may influence how they experience and frame interactions with people from different backgrounds, and thus, should have been collected and reported.

### Recommendations

Based on our findings, the following recommendations may help inform the normalisation of equitable referrals to the NHS LCD (and similar) programmes:

1. The LCD programme may achieve greater coherence (i.e. make greater sense) to some referrers by increasing promotional activities directed at healthcare professionals and by increasing opportunities for training. Furthermore, these promotional activities and training need to account for the different professional groups of referrers to the programme and thus their different needs, as has been recommended elsewhere [52].
2. Training should cover a broad range of issues that support the normalisation of referrals into routine care in equitable ways. For example, in addition to addressing common areas of need, such as confidence in deprescribing, training should seek to specifically support equitable, inclusive care for high need local populations, and may include for example, unconscious bias training.
3. Localities may also deploy more equitable approaches to the identification of patients. Alongside opportunistic referrals, system searches, alerts and prompts provide an opportunity to identify unmet need more proactively; incorporating these into training may also reduce the variation, and thus threats to coherence.
4. Supporting practices and referrers with resources, so that the additional workload associated with referral is not deprioritised amongst competing pressures, is likely to facilitate cognitive participation and collective action relating to the referral process.
5. As far as feasible, local health systems, including providers should support the referral and delivery in languages other than English where need arises, whilst also ensuring the cultural competence of the programme is appropriately addressed. This may contribute not only to greater equity in referral but also improve experience, retention and outcomes for non-English speakers on the programme.

## Conclusion

There remains a need to address the prevalence of T2D and obesity whilst also tackling health inequalities. LCD programmes achieved by TDR show promise as a viable, clinically effective, and cost-efficient treatment approach. Perhaps unsurprisingly given the timing of the LCD programme launch and our study data collection, our findings show that referral generation activities at that time had not yet been consistently normalised into routine care, a key step for establishing the LCD programme within standard practice in primary care. However, our findings also suggest that the LCD programme runs the risk of normalising an inequitable referral process. To avoid the occurrence of intervention-generated inequalities, it is important that an equitable referral process is normalised at a service delivery level. We have therefore made several recommendations to support the programme with achieving a more consistent normalisation of referrals to the LCD programme, with consideration of how to optimise greater equity in identification, referral, and service delivery.

### Supplementary Information

**Additional file 1.**

**Additional file 2.**

**Additional file 3.**

## Data Availability

The datasets generated during this current study are not publicly available due to reasons of privacy and confidentiality, and because of the inability to de-identify the data. Additional knowledge of the data can be available from the corresponding author on reasonable request.

## Abbreviations

T2D: Type 2 Diabetes
NHS: National Health Service
TDR: Total Diet Replacement
LCD: Low Calorie Diet Programme

## Footnotes

1 Integrated care systems are partnerships between NHS bodies, local authorities, and local organisations which work together on health and care services to improve the lives of people locally.

2 DESMOND – which stands for diabetes education and Self-management for ongoing and newly diagnosed – is a structured education programme for people with type 2 diabetes.
