# Supplementary material for: Normalisation and equity of referral to the NHS Low Calorie Diet programme pilot; a qualitative evaluation of the experiences of health care staff
Source: BMC Public Health. 2024 Jan 11;24:152. doi: 10.1186/s12889-023-17526-2 (PMC10782747; doi:10.1186/s12889-023-17526-2)
Supplement: Supplementary file 1 — Additional file 1. [file 12889_2023_17526_MOESM1_ESM.docx]

**Additional File 1**

# Overview of the first 10 localities commissioned by NHS E

**Table S1. Localities and delivery models of first 10 pilot sites**

| **Localities** | **Delivery Model** |
| --- | --- |
| Bedfordshire, Luton and Milton Keynes | Digital |
| Birmingham and Solihull | Group |
| Derbyshire | Group |
| Frimley | 1:1 |
| Gloucestershire | Digital |
| Greater Manchester | Group |
| Humber Coast and Vale | Digital |
| North Central London | Digital |
| North East London | Group |
| South Yorkshire, and Bassetlaw | 1:1 |


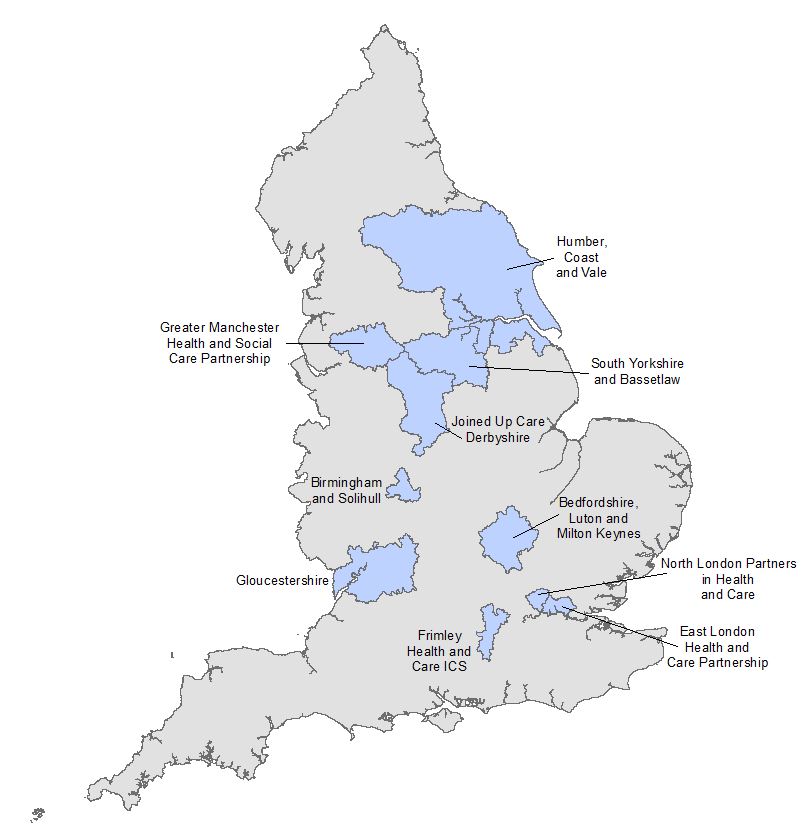


Geographical locations of the ten pilot sites; updated to April 2021 Integrated Care System configurations: taken from the Strategic Health Asset Planning Evaluation tool (SHAPE)

## Ethnicity

This section provides an overview of ethnicity across the ten pilot sites. Figures in the table below are presented as percentages (1).

**Table S2. Ethnicity at pilot site level**

| **Pilot sites** | **Asian / Asian British** | **Black / African/ Caribbean / Black British** | **Mixed / Multiple ethnic group** | **White** | **Other ethnic group** |
| --- | --- | --- | --- | --- | --- |
| South Yorkshire and Bassetlaw | 4.49 | 1.8 | 1.5 | 91.15 | 1.06 |
| Frimley | 13.41 | 2.77 | 2.27 | 80.55 | 1.01 |
| Greater Manchester HSCP | 10.03 | 2.73 | 2.25 | 83.97 | 1.01 |
| Joined Up Care Derbyshire | 4.02 | 1.01 | 1.41 | 93.12 | 0.43 |
| Birmingham & Solihull | 20.07 | 5.95 | 3.95 | 68.37 | 1.66 |
| East London Health and Care Partnership | 26.87 | 14.31 | 4.42 | 51.47 | 2.92 |
| Humber Coast and Vale | 1.76 | 0.49 | 0.92 | 96.49 | 0.34 |
| Gloucestershire | 2.08 | 0.86 | 1.45 | 95.42 | 0.18 |
| Bedfordshire, Luton, Milton Keynes | 12.44 | 5.39 | 3.08 | 78.34 | 0.75 |
| North London Partners in Health and Care | 13.29 | 12.83 | 5.67 | 63.7 | 4.51 |

## Deprivation

This section provides an overview of deprivation across the ten pilot sites, with data provided at a pilot site and CCG level. Table 3 indicates if the ‘Index of Multiple Deprivation’ (IMD) score for each pilot site, based on 2019 figures, is higher or lower than the average for England (21.67) (2).

**Table S3. Pilot Site IMD Score and its relation to the national average**

| **Pilot Sites** | **Higher/Lower than the National Average** |
| --- | --- |
| South Yorkshire and Bassetlaw | 28.38 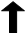 |
| Frimley Health and Care | 12.32 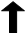 |
| Greater Manchester HSCP | 29.86 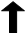 |
| Joined Up Care Derbyshire | 20.44 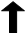 |
| Birmingham and Solihull | 33.59 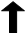 |
| East London Health and Care Partnership | 25.84 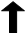 |
| Humber Coast and Vale | 20.91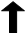 |
| Gloucestershire | 14.93 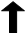 |
| Bedfordshire, Luton and Milton Keynes | 18.20 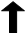 |
| North London Partners in Health and Care | 22.98 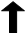 |

Table 4 provides an average IMD for each CCG across the 10 pilot sites. The ‘IMD average score’ is a population weighted average for LSOAs. The higher the average the greater the level of average deprivation (2).

**Table S4. IMD summary at a CCG level**

| **Pilot sites** | **CCGs (2021)** | **IMD Average Score (2019)** |
| --- | --- | --- |
| South Yorkshire and Bassetlaw | NHS Barnsley | 29.93 |
|  | NHS Bassetlaw | 22.59 |
|  | NHS Doncaster | 30.29 |
|  | NHS Rotherham | 29.55 |
|  | NHS Sheffield | 27.06 |
| Frimley Health and Care | NHS Frimley | 12.32 |
| Greater Manchester | NHS Bolton | 30.69 |
|  | NHS Bury | 23.68 |
|  | NHS Heywood, Middleton, Rochdale | 29.40 |
|  | NHS Manchester | 40.00 |
|  | NHS Oldham | 33.15 |
|  | NHS Salford | 34.21 |
|  | NHS Stockport | 20.83 |
|  | NHS Tameside and Glossop | 39.54 |
|  | NHS Trafford | 16.09 |
|  | NHS Wigan Borough | 25.71 |
| Joined Up Care Derbyshire | NHS Derby and Derbyshire | 20.44 |
| Birmingham and Solihull | NHS Birmingham and Solihull | 33.59 |
| East London Health and Care Partnership | NHS North East London | 25.84 |
| Humber Coast and Vale | NHS East Riding of Yorkshire | 16.08 |
|  | NHS Hull | 40.56 |
|  | NHS North East Lincolnshire | 31.34 |
|  | NHS North Lincolnshire | 22.13 |
|  | NHS North Yorkshire | 15.63 |
|  | NHS Vale of York | 11.90 |
| Gloucestershire | NHS Gloucestershire | 14.93 |
| Bedfordshire, Luton, Milton Keynes | NHS Bedfordshire, Luton and Milton Keynes | 18.20 |
| North London Partners in Health and Care | NHS North Central London | 22.98 |

## Urbanity and Rurality

This section provides an summary of the classifications on urbanity and rurality across the ten pilot sites (see Table 5) (3). The rural/urban classification, is classified based on the categorisation of lower super output areas (LSOAs). LSOAs are geographical areas generated to have similar population sizes. They have a minimum population of 1000 and a mean of 1500, covering approximately 650 households.

**Table S5. Rural/Urban Classification for the ten pilot sites**

| **Pilot Sites** | **Rural Classification for the ten Pilot sites** |
| --- | --- |
| South Yorkshire and Bassetlaw | Predominantly a mix of rural village and dispersed and urban minor conurbation |
| Frimley Health and Care | Predominantly urban city and town in a sparse setting |
| Greater Manchester HSCP | Predominantly urban major conurbation |
| Joined Up Care Derbyshire | Predominantly rural village and dispersed and urban city and town in a sparse setting |
| Birmingham and Solihull | Predominantly urban major conurbation |
| East London Health and Care Partnership | Predominantly urban major conurbation |
| Humber Coast and Vale | Predominantly rural village and dispersed and rural village and dispersed in a sparse setting, with some urban city and town in a sparse setting |
| Gloucestershire | Predominantly rural village and dispersed and urban city and town in a sparse setting |
| Bedfordshire, Luton and Milton Keynes | Predominantly rural village and dispersed and urban city and town in a sparse setting |
| North London Partners in Health and Care | Predominantly urban major conurbation |

# The NHS Low Calorie Diet Programme Delivery Structure


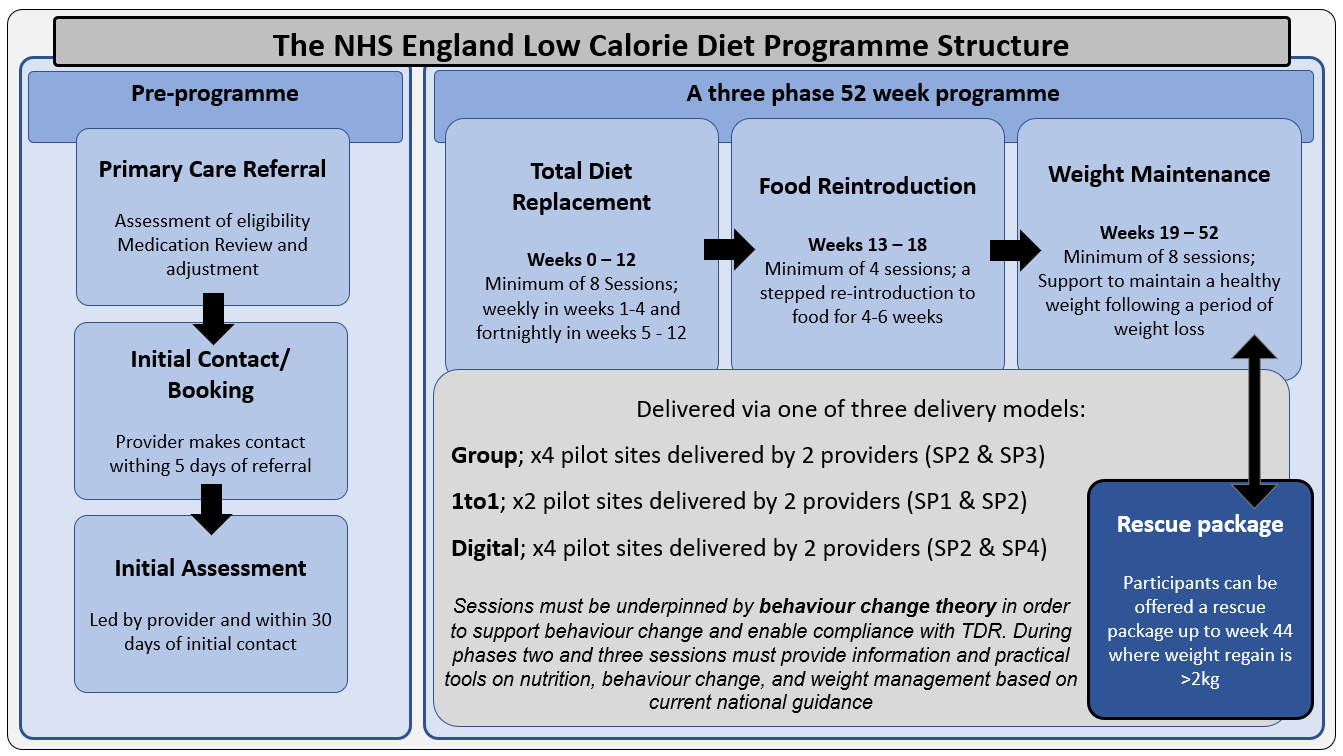


**Figure S1. The NHS Low Calorie Diet Programme Delivery Structure**

The four providers used different TDR product brands with large difference in range of products and flavours available. One provider provided 6 different options (soups and shakes) while a second provided 89 different options (soups, shakes, smoothies, bars, breakfasts, and pre-prepared meals). The other two providers provided 15 (soups, shakes, smoothies, and porridge) and 7 (soups, shakes and bars) options respectively.

**Table S6. Job and referral status of referrers**

| **Participant Identification** | **Referral status** | **Job** |
| --- | --- | --- |
| R1 | Low | GP |
| R2 | High | Advanced Nurse Practitioner |
| R3 | Low | Practice Nurse |
| R4 | High | GP |
| R5 | High | Practice Nurse |
| R6 | High | Practice Nurse |
| R7 | High | Practice Nurse |
| R8 | Low | Practice Nurse |
| R9 | High | GP |
| R10 | High | Pharmacist |
| R11 | High | Practice Nurse |
| R12 | Low | Practice Nurse |
| R13 | Low | GP |
| R14 | Low | Pharmacist |
| R15 | Low | Practice Nurse |
| R16 | High | GP |
| R17 | High | GP |
| R18 | Low | Practice Nurse |
| R19 | Low | Practice Nurse |
| R20 |  |  |

# References

1. Ethnicty [Internet]. Office for National Statistics. 2011 [cited 22nd June 2021]. Available from: <https://www.ons.gov.uk/peoplepopulationandcommunity/culturalidentity/ethnicity>.

2. English Indices of Deprivation 2019 : File 13 - Clinical Commissioning Group (CCG) summaries [Internet]. 2019 [cited 19th March 2021]. Available from: <https://www.gov.uk/government/statistics/english-indices-of-deprivation-2019>.

3. 2011 rural/urban classification [Internet]. Office for National Statistics. 2011 [cited 22nd June 2021]. Available from: <https://www.ons.gov.uk/methodology/geography/geographicalproducts/ruralurbanclassifications/2011ruralurbanclassification>.
