# Supplementary material for: Normalisation and equity of referral to the NHS Low Calorie Diet programme pilot; a qualitative evaluation of the experiences of health care staff
Source: BMC Public Health. 2024 Jan 11;24:152. doi: 10.1186/s12889-023-17526-2 (PMC10782747; doi:10.1186/s12889-023-17526-2)
Supplement: Supplementary file 3 — Additional file 3. [file 12889_2023_17526_MOESM3_ESM.docx]

**Additional File 3**

# Covid 19 and the demands on Primary Care (theme 1)

“The other thing that I think is a barrier that I've heard from other GPs but not witnessed myself in terms of workload, is that they're really quite cautious over the idea of an increased workload if somebody joins the programme and then continues to have high sugar readings for example, or low blood pressure readings or high blood pressure readings, and then the provider sends clinical alerts to the surgery that then requires an urgent review, inevitably that goes on to the bottom of a duty doctors list, which is invariably very busy. And I'm afraid to say that GPs, because are chronically overburdened, anything that then might pose a slight increase in workload, even if it doesn't actually turn out to be true, meets a lot of resistance” (R1 – GP).

“I’d probably say it’s the time. So I think because of the pandemic we’ve lost a lot of administration time and we see a lot more patients that would have been seen in hospital or there’s lots of things happening that wouldn’t have happened normally. So because people can’t have their vaccinations in hospital or because their babies can’t have their vaccination that they needed at four weeks we’re then sort of seeing people, a lot more people that have to be seen on sort of a timely schedule. So that sometimes means that we have to see people in our administration time, and it doesn’t leave a lot of time to sort of action this programme” (R5 – Practice Nurse).

“It's a combination of premises, workload, prioritisation, all the other things that have come in, and the backlog. And there is a huge backlog, and a lot of the backlog is patients waiting for hospital treatment who are either, you know, sort of in more pain or moaning because they haven't been contacted by the hospital and expecting us to chase things so it, so it has meant that just even in your day you're weary. And trying to then have a consultation that about the low calorie diet or any diet weight management program as an individual unless you've got some passion, I think that it's quite easy to just do the bit, to do the basics, to do the, to do that sort of bare minimum firefighting” (R9 – GP).

“I think our limitation has been the time and the also the, the NHS work pattern at the moment in terms of the demands for other reasons in terms of the vaccinations and in terms of the increased COVID cases and the respiratory infection rates, etc” (R13 – GP).

“We’ve certainly seen a trend with our results with HbA1cs you know people who’ve been controlled for many years suddenly being not as well controlled purely because of lockdown being working from home, less exercise and motivation” (R11 – Practice Nurse).

# The expertise and knowledge of referral staff (theme 2)

“As I mentioned it just about how the patients will engage the program and what will happen at every stage. More was needed so that we can then pass on to the patient when we're trying to recruit them 'cause they, they ask how it will work” (R10 – Pharmacist).

“I think it had all the information there, but I think because the referral form was so bulky I think initially it was very overwhelming. So we were trying to refer patients, but then I think a couple of referrals got bounced back” (R5 – Practice Nurse).

“Yes, because when it was first being, you know, we were told that it was coming out into our area so yes, so there were video sessions that we had, there was emails that were sent out, one of the local GPs who's heavily involved also sort of did a tutorial on it as well for some of the referral aspects to it” (R7 – Practice Nurse).

“My only criticism of the programme is what happens after, there's very little or, you don't, it feels like you don't get to find out much about what happened you know. I think with some of them I was expecting better results than what came through. Yeah, I think the communication at the end is very basic. Whether that could be a bit more in depth would be probably useful” (R15 – Practice Nurse).

“I'm not an email person and I think some Zoom or some Teams meetings would be good just to sort of, so that we're all on the same page and we're all being told the same thing. But at the moment it's bits and pieces” (R6 – Practice Nurse).

“I think, I think there possibly could be a bit more information and I think from a training perspective I think there was at one point a session offered for sort of more discussion about it but I think at the time I, it had only just come on to our radar and I think we had all sorts and everything else going on as well and so I just wasn’t able to do that session. So maybe a couple more sessions of that sort of for people so that other people can sort of learn about it and know what they’re looking for and doing with it” (R18 – Practice Nurse).

“I felt absolutely fine. I felt yeah, I felt well prepared to do it, yeah” (R7 – Practice Nurse).

“I suppose this is where I declare that I'm actually the [area] clinical lead, the [area] clinical lead, so the responsibility for some of the training was on me. So yes, I received the information and worked really quite closely with our provider to put on a webinar and then and sort of to agree that training and sort of what that would look like, you know. So I'd had a slightly different perspective. I think that from my point of view that that training was really needed, so I needed to go through and understand the process so that I could then share that with others” (R9 – GP).

# Patient Identification and the referral process (theme 3)

“The best way to pick them up really at a diabetic review as well” (R8 - Practice Nurse).

“Those that don't come for their yearly reviews and you have to really chase them for three or four appointments. And they have no control over their diabetes, they're overweight, they're eating the wrong food, they don't exercise, their diabetes has spiralled out of control. Those are the ones that you really want them to engage in something like this, and they’re most likely the ones that don't” (R6 - Practice Nurse).

“If you are head of diabetes in the practice, you tend to know your patients better so you know who will be interested, who has already been interested, who already knows about similar programmes” (R13 - GP).

“So obviously all diabetics have at least two reviews a year, six monthly, or three monthly if they’re out of target. So those are all opportunities to bring something like this up. So that, that's really good. It's just whether you're familiar with it or not, so I am, other people may not be” (R2 – Advanced Nurse Practitioner).

It's a mixture isn’t it, it’s about so I got a, no so I did do a list. So initially I started with a list so and this was generated meeting the criteria. […]. So getting the list, but also because if you head diabetes in the practice, you tend to know your patients better so you know who will be interested, who has already been interested, who already knows about similar programmes. So that’s how I did my ones (R13 – GP).

“So when we first, when we first started it we did a search basically for all our diabetic patients and sent them all out a text to say right, this is what’s starting in the area, let us know if you're interested. So, so a lot came through that way initially, then since then it's generally when we've been doing reviews” (R7 – Practice Nurse).

“I've had two referrals that I've sent, and that's when I've realised that they've bounced back 'cause I've missed something. So yeah, sadly, I've only got one story that I can kind of say that has gone through and that patient was super keen and on board and very grateful for the referral” (R12 – Practice Nurse).

“Mostly those that are non attenders, those that don't come for their yearly and you have to really chase them for three or four appointments. And they have no control over their diabetes, they're overweight, they're eating the wrong food, they don't exercise, their diabetes has spiralled out of control. Those are the ones that you really want them to engage in something like this, and they’re most likely the ones that don't” (R6 – Practice Nurse).

# Barriers to referrals (theme 4)

“So there is an element of the time consuming part that is a little bit of a barrier, but it doesn’t stop me from doing it. It’s just I kind of need to use my admin time to do it and so it’s not something I can just pick up and do for five minutes and then put down again. It’s something that you kind of, I have to get out in front of me so that I can sort of check through the criteria with each person” (R18 – Practice Nurse).

“it really gets me down because I'm really passionate about this very low calorie diet. So I think for me I would say following the last six months in my practice particularly, we've lost something like six nursing staff” (R8 – Practice Nurse).

“All that will, can then be passed back to other patients who will probably benefit. Barriers I think the main issue after discussing with some of my neighbouring GP practices colleagues was that there was sometimes there is no proper supervision from a GP lead and it's all left to the practice nurses. And then no one is checking that how many referrals have been done or not done so then the practice nurses are then just waiting for the diabetes annual review to turn up and then referring rather than being proactive in looking into it” (R4 – GP).

“But obviously then COVID struck and then we didn't really see face to face. So I think in the past year it's only, it's only been the odd one or two that I’ve, we've referred because we literally haven't had that time, which it sounds awful, but that's what's happened I would say” (R8 – Practice Nurse). .

“sometimes I think people can get a bit set in their ways about how we manage patients and that these, you know these things might not necessarily be successful, so let's just throw medication at them so you know.” (R15 – Practice Nurse).

“We get really bombarded and overloaded by different schemes so I should think one of the barriers would be for obviously I, I work for the CCG, and I did the webinar for this, so I'm very familiar with it, but for those that aren't, they would perhaps open the template or the form and think, oh my goodness, a bit overwhelming and they may not then proceed if they’re less familiar so, because time is of the essence always” (R2 – Practice Nurse).

“I think she probably could have got through it without throwing away the principles of the whole project but being able to say, the dietician saying that soup that you’ve chosen does look relatively similar to my one, have the whatever flavour that we don’t have available. So I think that’s something that the provider should be thinking on about and the commissioner should be thinking about is there room for flexibility and my sense is for some people there should be a bit more room than there has been” (R16 – GP).

# Who gets referred to the LCD programme (theme 5)

“There's also I think because our demographics in inner city [area] the language barrier is the fact that we are very large Asian population, a lot of older generation grasp of English is very basic” (R10 - Pharmacist).

“Culturally it's not accepted you know, to replace meals like that. Their Asian diet is, staple diet is eating rice at each meal, and then you're saying, you know, and they’ve come to England and they've adopted our, our diet and then then all of a sudden, they've been told that they’ve diabetes and they're overweight and they need to have replacement meals. I don't think it's acceptable for them” (R6 - Practice Nurse).

“Or they were not referred because we, I just thought that they don't have any language support so it may not be the best option for them. Not that they're refusing” (R13 - GP).

“Going back to the Nepalese population, I find it difficult sometimes to even have a telephone conversation with them because they often rely on a translator that I need to talk through, like another family member. So then for me to then start offering other things like that, I probably would just avoid it because I know how difficult it is for me to communicate with them. They’re often not answering their phone and I’m having to chase them up, so I know that they're not going to be committed to it. […] it would put me off referring them because I would feel like they're, they're not going to be committed” (R12 - Practice Nurse).

“If I, if I'm honest, I would say that the, the patients who are from the sort of the, the South Asian ethnic sort of population, that was a huge barrier because of, it's quite an intense programme. […]. So you're looking at just soups and shakes, and so you're, you're asking these people who food is very much part for these, it's, it's more like a cultural thing, really, food there and the way the food is prepared, which is really sort of plays a huge part also in diabetes as well” (R14 – Pharmacist).

“Because you've got staff who haven't got the time to devote to these kind of things in areas of deprivation, because there's usually less staff in deprived areas because people don't want to work in them, and they don't get remunerated in the same way as in some of the less deprived areas” (R2 – Advanced Nurse Practitioner).

“It is generally the younger population would speak English across the board unless they’re new migrants, new to the country. Then they, so yeah, so if it’s face to face for elderly patients they maybe do something. I think the fact that if it’s telephonic or video that's not something that they are comfortable, comfortable with. And the online, also the fact that there’s online classes or there’s classes that they need to attend to that can put early patients off because yeah” (R10 - Pharmacist).
